# Application Potential of *CTHRC1* as a Diagnostic and Prognostic Indicator for Colon Adenocarcinoma

**DOI:** 10.3389/fmolb.2022.849771

**Published:** 2022-03-01

**Authors:** Chen Pang, Hongwei Wang, Chengcheng Shen, Houjie Liang

**Affiliations:** ^1^ Department of Oncology and Southwest Cancer Centre, Southwest Hospital, Third Military Medical University (Army Medical University), Chongqing, China; ^2^ Department of Dermatology, The First Affiliated Hospital of Chongqing Medical University, Chongqing, China

**Keywords:** collagen triple helix repeat containing 1 (CTHRC1), colon adenocarcinoma (COAD), diagnosis, prognosis, immune infiltration, function pathway

## Abstract

Colon adenocarcinoma (COAD), ranking third in incidence and second in mortality, is one of the most common cancer types in the world. The initial stages of COAD usually show no obvious clinical symptoms; moreover, effective screening or diagnostic indicators with high sensitivity and specificity are lacking, which often leads to missed treatment opportunities. Collagen triple helix repeat containing 1 (*CTHRC1*) is a glycosylated protein secreted during tissue repair, which reduces collagen matrix deposition and promotes cell migration. Under physiological conditions, the expression of *CTHRC1* is conducive to wound healing; however, the pathological overexpression of *CTHRC1* promotes tumour growth and proliferation. In this study, we evaluated the application potential of *CTHRC1* as an early diagnosis and prognostic survival monitoring biomarker for COAD in addition to unravelling its molecular mechanism in the development of COAD and exploring new therapeutic targets. Therefore, various tumour databases were used to investigate the expression of *CTHRC1* in COAD at the mRNA and protein levels. *CTHRC1* expression was found to be significantly increased in COAD, regardless of clinical cancer stage, age, sex or race. Moreover, *CTHRC1* expression was significantly correlated with poor prognosis and positively correlated with CD8^+^ T cell, CD4^+^ T cell, neutrophil, macrophage and dendritic cell infiltration. The relevant function pathways and neighbouring proteins to *CTHRC1* in COAD were identified as ROR2, VAPA, LY6E and several collagen family proteins. Therefore, this study suggests that *CTHRC1* is a potential diagnostic and prognostic biomarker for patients with COAD.

## Introduction

A recent study by the American Cancer Society reported that colorectal cancer has the third highest (10.0%) incidence rate after female breast cancer (11.7%) and lung cancer (11.4%) (Sung et al., 2021). The incidence rate of colorectal cancer in developed countries is approximately four times higher than that in developing countries (Fidler et al., 2016). Based on the current prediction models, the global incidence of colorectal cancer has been estimated to reach approximately 2.2 million new cases/year by 2030, accounting for 20% of all patients with cancer (Araghi et al., 2018). Colorectal carcinoma has the second-highest death rate of 9.4% after lung cancer. The official statistics on the prognosis of patients with colorectal cancer published by the American Cancer Society report that the 5 year survival rate is approximately 64% (Sung et al., 2021). Specifically, early diagnosis or detection of colorectal cancer at stage I, stage IIA or IIB increases the 5 years survival rate to approximately 90%; however, delayed diagnosis decreases the survival rate of patients with pathological stage IV tumour to approximately 14% (Pilonis et al., 2020). Although the current screening of colorectal cancer has been strengthened, resulting in a slight decrease in late diagnosis case numbers, up to 90% of cases are diagnosed after symptoms appear (Vanessa and Karen, 2016).

The occurrence of colorectal cancer is attributed to complex genetic and environmental factor interactions, involving multiple genes at different stages. The main pathways include the chromosomal instability pathway, CpG island methylator phenotype pathway, microsatellite instability pathway and the serrated pathway (Harrison and Benziger, 2011). Colon adenocarcinoma (COAD) is the main manifestation of colorectal cancer, with more than 80% of colorectal cancers being diagnosed as COAD (Li and Gu, 2005). The initial stage of COAD usually shows no obvious clinical symptoms, and the lack of effective screening or highly sensitive and specific diagnostic indicators often lead to missed treatment opportunities (Garborg et al., 2013). Therefore, it is crucial to establish an effective screening mechanism to improve the early diagnosis rate.

Collagen triple helix repeat containing 1 (CTHRC1) protein was first identified in the injured arteries of rats as an extracellular secretory protein, which is expressed in the injured part and smooth muscle cells of neointima, mainly promoting the growth and proliferation of newly generated cells (Leclair and Lindner, 2007). CTHRC1 regulates the occurrence and development of cervical, pancreatic and liver carcinoma by participating in cell proliferation, cell migration, type I collagen synthesis and damaged vascular repair (Tameda et al., 2014). Studies have shown that promoting CTHRC1 expression increased the migration and invasion of primary gastrointestinal stromal tumour cells, whereas silencing CTHRC1 expression inhibited the epithelial–mesenchymal transformation of glioblastoma cells (Ma et al., 2014). CTHRC1 promotes the proliferation of colorectal cancer by activating the Wnt/PCP signalling pathway (Yang et al., 2015). Additionally, CTHRC1 plays an important role in the pathogenesis of systemic lupus erythematosus and other diseases (Wu et al., 2018).

This study, therefore, aimed to evaluate the application potential of CTHRC1 as an early diagnosis and prognostic survival monitoring biomarker for COAD. Additionally, the molecular mechanism of COAD occurrence and development along with various novel therapeutic targets were explored. Therefore, the expression of CTHRC1 in COAD at the mRNA and protein levels was investigated using various tumour databases. By evaluating the expression of CTHRC1 in patients with COAD under different physiological and pathological conditions, the application potential of CTHRC1 as a diagnostic indicator was determined. Moreover, the effects of different expression levels and genetic mutations of *CTHRC1* on COAD survival rate was analysed, including the immune infiltration of CTHRC1 in COAD. Further, the associated proteins and pathways of CTHRC1 in tumorigenesis are also discussed.

## Materials and Methods

### Oncomine Analysis

The Oncomine platform (https://www.oncomine.org/) is a publicly accessible, online tumour-related gene microarray database that collects disease-related gene expression profiles and relevant clinical information. The expression level of *CTHRC1* in different cancers was investigated *via* Oncomine. When compared to corresponding normal tissues, the transcriptional levels were considered statistically significant at fold change >1.5 and *p*-value < 0.001. The threshold value of gene rank was set to “top 10%”, and the data type was set to “mRNA” (Rhodes et al., 2004).

### TIMER 2.0 Analysis

TIMER 2.0 (http://timer.comp-genomics.org/) was employed to investigate the expression levels of *CTHRC1* in various tumour tissues. Additionally, data of 32 tumour types from more than 10,000 samples were collected from the TCGA database and used for immune infiltration analysis via TIMER 2.0, which ascertains the abundance of tumour infiltrates based on gene expression levels. *CTHRC1* was chosen as the input and tumour cells were detected under the Immune Association module. B cells, CD8^+^ T cells, CD4^+^ T cells, neutrophils, macrophages and dendritic cells were selected as the test types based on the study by Li et al. and Danaher et al. (Li et al., 2016; Danaher et al., 2017). Gene expression values were converted to Log2 RNA-Seq by Expectation-Maximization values.

### Human Protein Atlas Analysis

The Human Protein Atlas (https://www.proteinatlas.org) is an online dataset that collects the expression characteristics of various functional proteins *via* immunohistochemistry from tumours and corresponding normal tissues (Asplund et al., 2012). The Human Protein Atlas was used to compare the expression of CTHRC1 proteins in normal and COAD tissues using the images of immunohistochemical staining.

### UALCAN Analysis

UALCAN (http://ualcan.path.uab.edu) is an open-access web platform that contains cancer-related clinical data, which can be obtained from the TCGA database. This was used to compare the *CTHRC1* expression levels between the COAD and normal tissues, along with the relationship between gene expression and pathologic features in these tissues (Chandrashekar et al., 2017). In the website, TCGA was chosen, and the corresponding tumour type was selected. The student’s *t*-test was used to compare the transcription levels of *CTHRC1* between the tissue types, and *p* < 0.05 was considered statistically significant.

### GEPIA Analysis

GEPIA (http://gepia.cancer-pku.cn/index.html) was used to analyse the relationship between *CTHRC1* expression and overall survival (OS) or disease-free survival (DFS) prognosis in patients with COAD based on the parameters of hazard ratios and log-rank *p*-values. After logging onto the database, Single Gene Analysis was firstly chosen. After entering *CTHRC1*, “survival plots” was selected. Survival analysis was performed using the following parameters: Group Cut-off: Median; Hazards Ratio: Yes; 95% Confidence Interval: Yes (Tang et al., 2017).

### cBioPortal Analysis

cBioPortal (http://www.cbioportal.org/) was used to analyse the alteration frequency of *CTHRC1* gene mutations. Putative copy-number calls on 478 cases were determined using GISTIC 2.0. In the module Comparison/Survival, the influence of the alterations on prognostic survival in patients with COAD was analysed using default parameters (Gao et al., 2013).

### Functional Analysis

GeneMANIA was used to identify the physical interaction and co-expression of CTHRC1 with 20 related proteins using the *Homo sapiens* datasets with default parameters (Mostafavi et al., 2008). GO enrichment and KEGG pathway analyses (FDR cutoff <0.05) of related gene were conducted using ShinyGO v0.741 (http://bioinformatics.sdstate.edu/go/).

## Results

### Expression of *CTHRC1* in COAD

**FIGURE 1:**
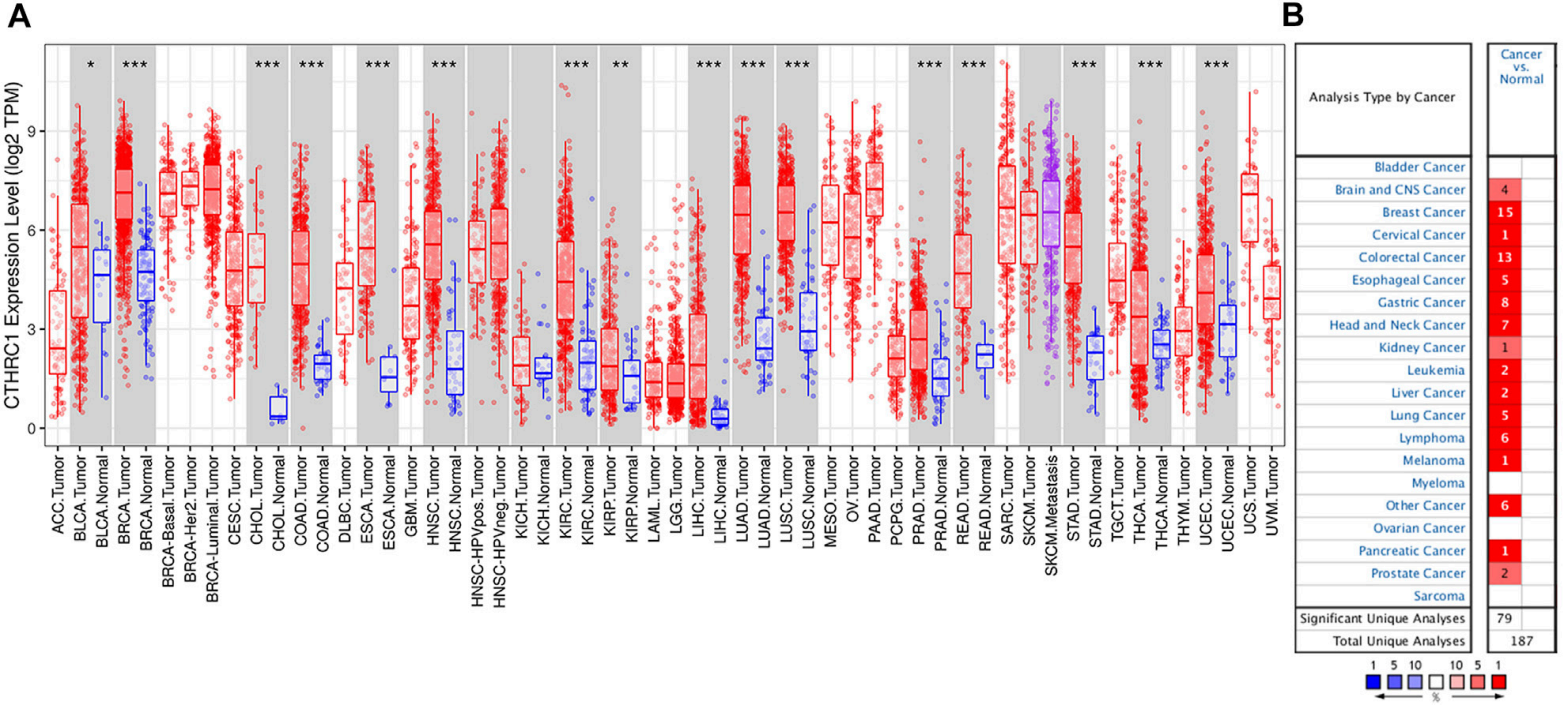
Expression levels of CTHRC1 in different types of tumour tissues. **(A)** *CTHRC1* expression in various tumour tissues *via* TIMER analysis. **(B)** *CTHRC1* expression in different cancer types *via* Oncomine analysis (*p < 0.05; **p < 0.01; ***p < 0.001).

**FIGURE 2:**
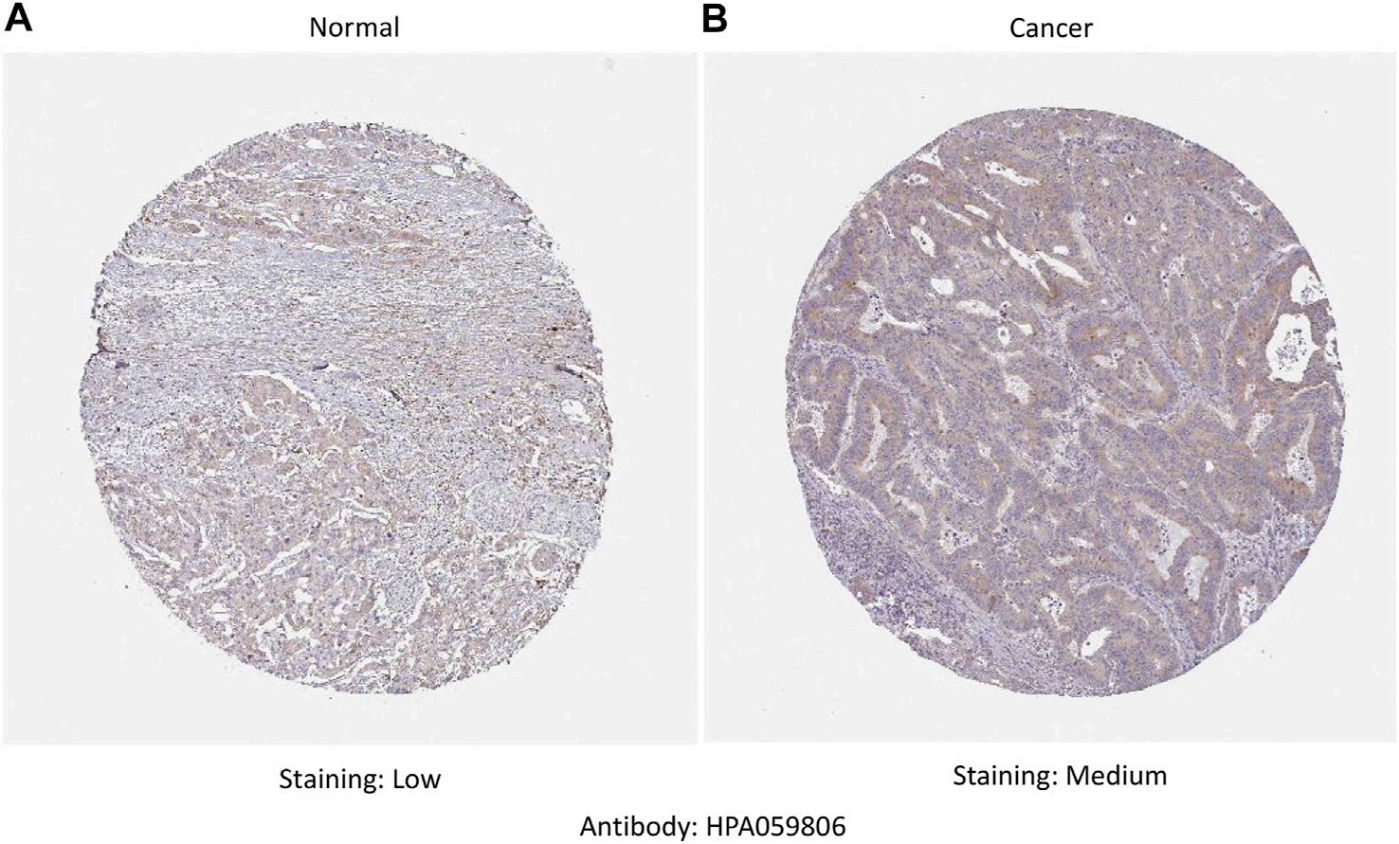
Analysis of CTHRC1 protein expression in colon adenocarcinoma tissue collected from the Human Protein Atlas dataset. **(A)** The control (normal) tissue with low staining; **(B)** The colon adenocarcinoma tissue with medium staining. Primary antibody: HPA059806.

RNA-seq data extracted from TCGA database showed a consistent trend of abnormally high *CTHRC1* expression in more than 16 types of tumour tissues compared with the corresponding normal tissues, such as COAD, breast invasive carcinoma and stomach adenocarcinoma (Figure 1A). Similarly, Oncomine analysis of the pathological samples showed that the transcriptional levels of *CTHRC1* mRNA were significantly up-regulated in various cancer types including colorectal cancer (Figure 1B). Further comparison of the expression levels of CTHRC1 protein between the normal and COAD tissue using immunohistochemical data from the Human Protein Atlas dataset showed that CTHRC1 protein expression in COAD was consistent with the mRNA detected (Figure 2). These findings strongly suggest the positive role of *CTHRC1* in COAD tumorigenesis.

### Expression of *CTHRC1* in Patients With COAD Under Different Physiological or Pathological States

**FIGURE 3:**
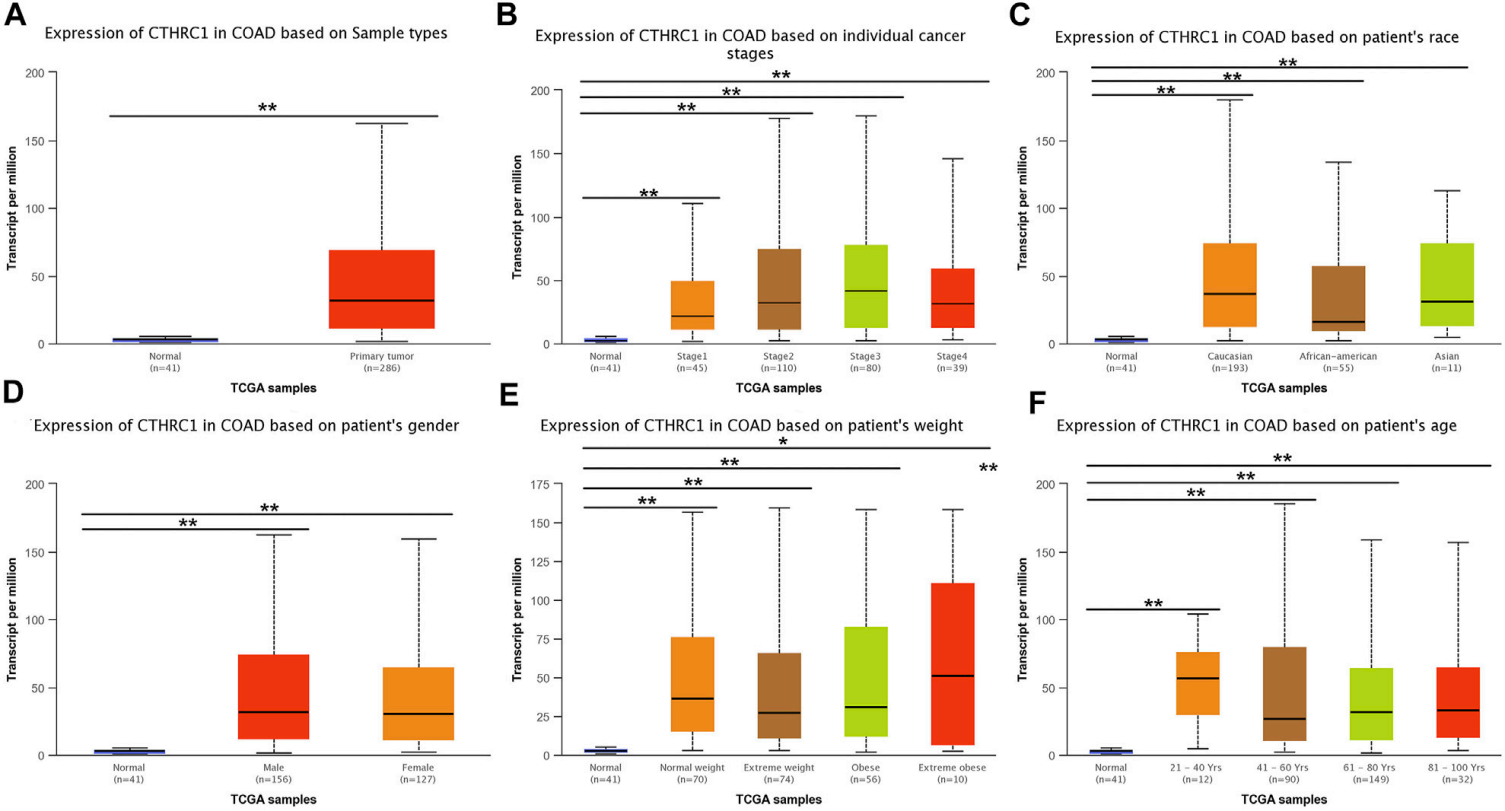
Analysis of *CTHRC1* expression in patients with colon adenocarcinoma (COAD) under different physiological or pathological states using UALCAN. **(A–F)** *CTHRC1* expression in COAD patients of various physiological and pathological states (^*^ denotes 0.01 < *p* < 0.05, ^**^ denotes *p* < 0.01).

UALCAN analysis showed that the expression level of *CTHRC1* was significantly higher in the patients with primary COAD than that in normal tissues (Figure 3A). Notably, this abnormally high expression pattern is generally applicable to patients with different clinicopathological characteristics, such as clinical cancer stage, age, sex or race. However, no significant difference in expression level was observed among patients diagnosed with different states, apart from the high expression in patients aged 21–40 years (Figures 3B–F). Importantly, *CTHRC1* showed a strong abnormal expression in patients with early stage COAD, *i.e.*, *CTHRC1* has shown significant high expression in stage I COAD, which substantiates the potential role of *CTHRC1* as an early diagnostic biomarker for COAD.

### Prognostic Value of *CTHRC1* Expression in Patients With COAD

**FIGURE 4:**
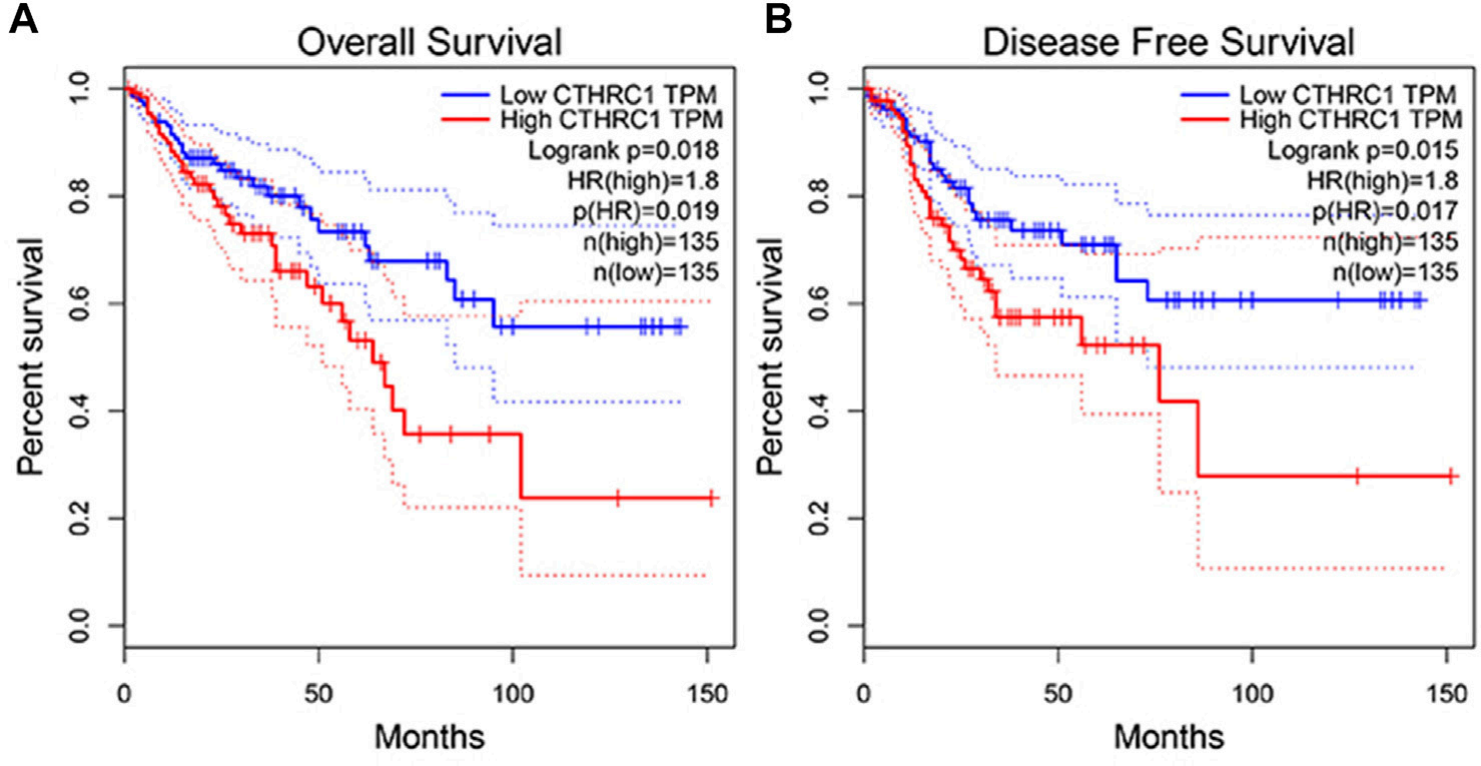
Correlation analysis between CTHRC1 expression and prognostic survival in patients with colon adenocarcinoma (COAD) using GEPIA. **(A)** Correlation analysis between *CTHRC1* expression and overall survival in patients with COAD; **(B)** Correlation analysis between *CTHRC1* expression and disease free survival in patients with COAD.

**FIGURE 5:**
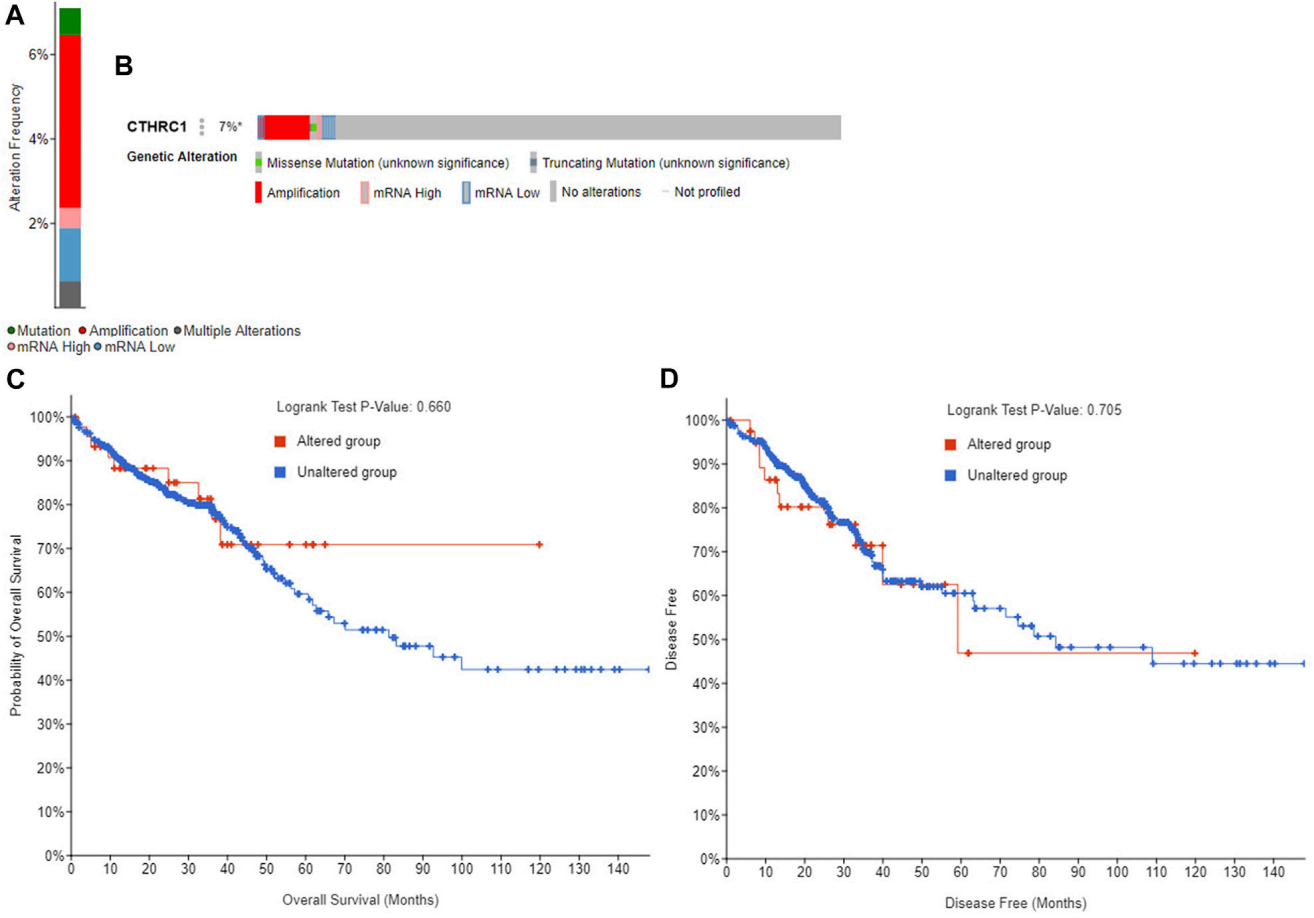
Alteration frequency analysis of *CTHRC1* and its influence on prognosis in patients with colorectal cancer using cBioPortal. **(A)** Alteration frequency of *CTHRC1* in patients with colorectal cancer. **(B)** Effect of *CTHRC1* alteration on the overall survival of COAD patients. **(C)** Effect of *CTHRC1* alteration on the disease free survival of COAD patients. **(D)** Effect of *CTHRC1* alteration on the disease free survival of COAD patients.

The GEPIA dataset was employed to assess the prognostic value of *CTHRC1* expression in patients with COAD. *CTHRC1* proves to be a promising prognostic indicator due to the high expression level of *CTHRC1,* which indicates poor OS and DFS prognosis with a same high hazard ratio of 1.8 (Figure 4). Investigation of the alteration frequency of *CTHRC1* using the TCGA database revealed approximately 7.1% gene alteration in 634 colorectal cancer cases (Figure 5A,B). However, these mutations did not significantly affect the OS and DFS of patients (Figure 5C,D), indicating that *CTHRC1* has considerable stability as a prognostic indicator.

### Correlation Analysis Between *CTHRC1* Expression and Immune Cell Infiltration in COAD Tissue

**FIGURE 6:**
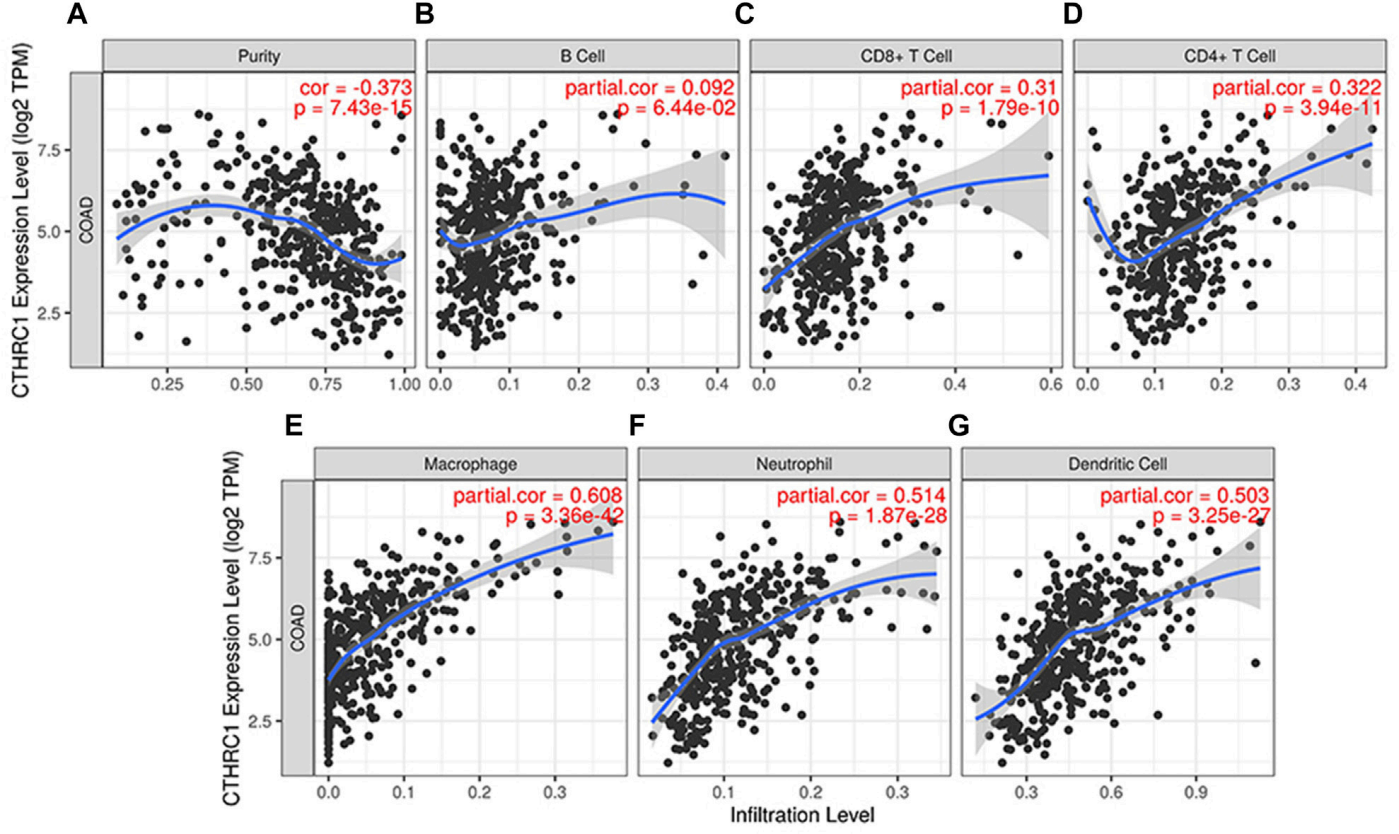
Correlation between *CTHRC1* expression and immune cell infiltration levels in colon adenocarcinoma tissue analysed via TIMER 2.0. **(A–G)** The correlation between *CTHRC1* expression and immune cell infiltration in COAD patients (*n* = 458).

TIMER 2.0 was used to analyse the correlation between *CTHRC1* expression and immune cell infiltration levels, including B cells, CD8^+^ T cells, CD4^+^ T cells, neutrophils, macrophages and dendritic cells. *CTHRC1* expression was significantly positively correlated with all the test immune cells except B cells (*p* = 0.0644). Among them, macrophages had the strongest correlation with *CTHRC1*, with a high partial correlation coefficient of 0.608 and a *p*-value of 3.36e^−42^ (Figure 6). To clarify the immune response mechanism induced by *CTHRC1* and develop new immunotherapeutic targets, the expression correlation between *CTHRC1* and immune cell subsets was investigated. *CTHRC1* was significantly correlated with most of the corresponding biomarker genes of the test immune cell subsets (Supplementary Table S1).

### Co-Expression and Interaction Analysis of *CTHRC1* in COAD Tissue

**FIGURE 7:**
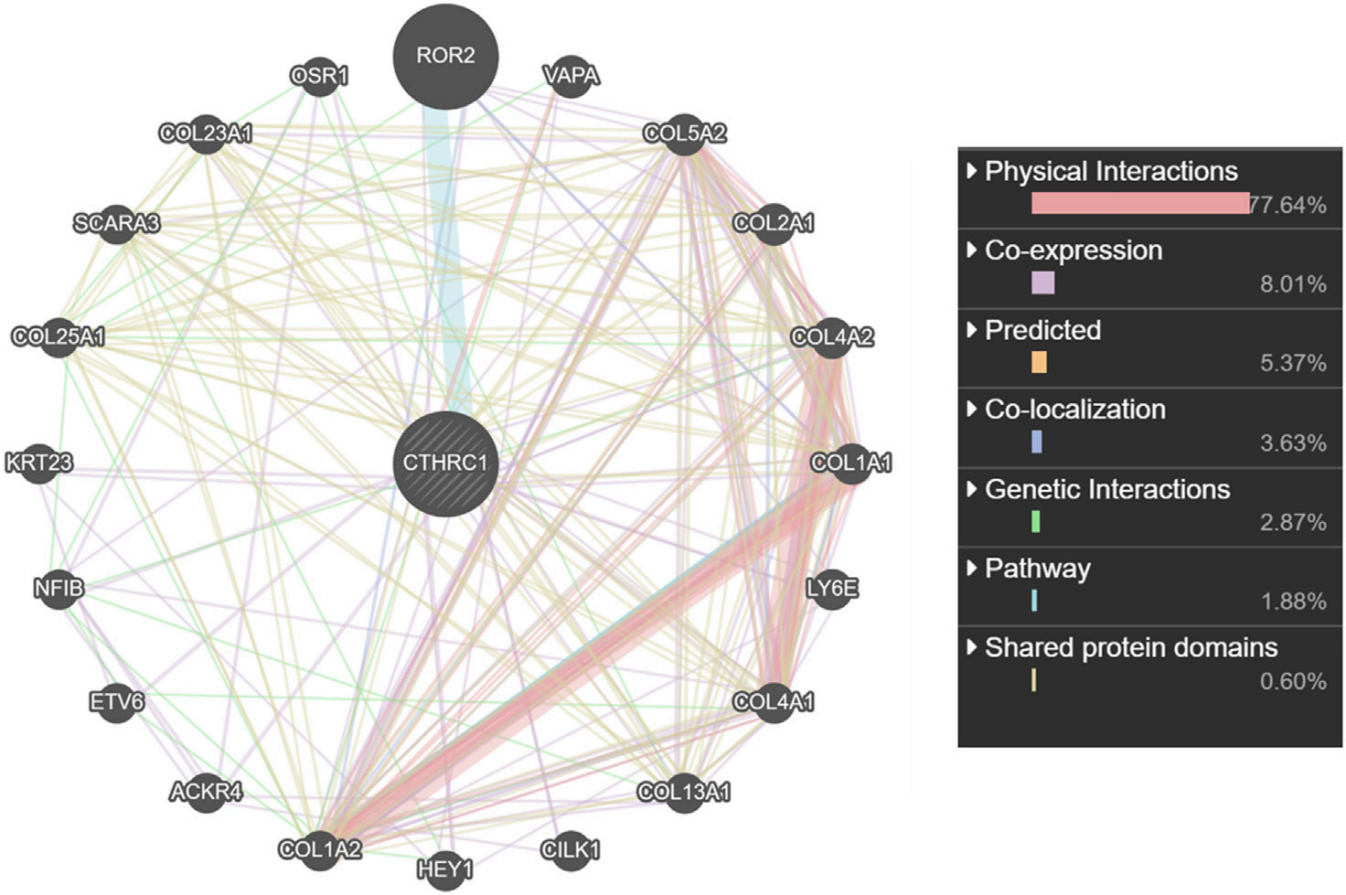
Interaction network analysis of CTHRC1 and its most similar proteins using GeneMANIA.

**FIGURE 8:**
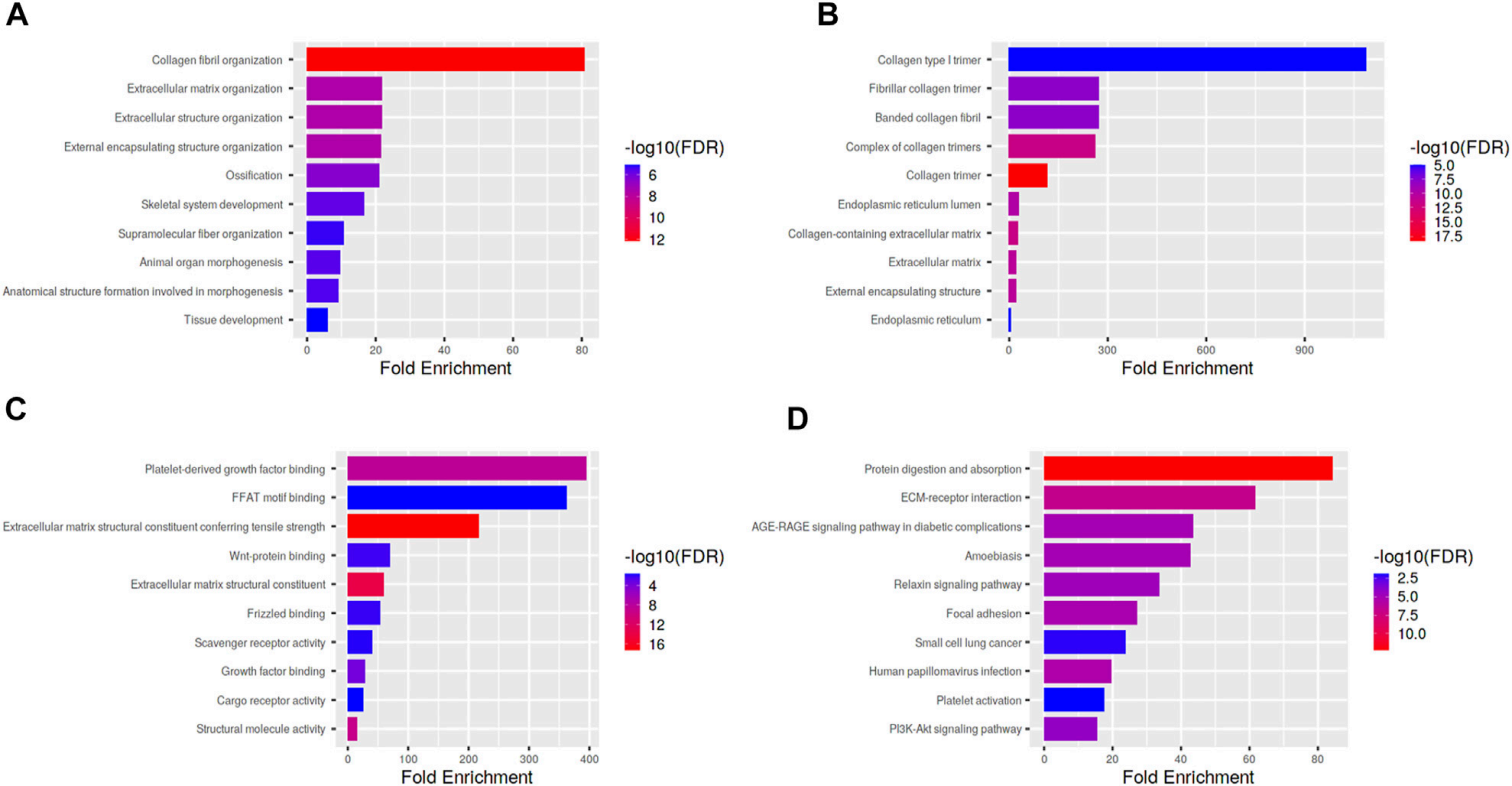
Enrichment analysis of the Kyoto Encyclopedia of Genes and Genomes functional pathways of CTHRC1 and their related proteins using ShinyGO. **(A)** GO enrichment analysis of CTHRC1 in biological process of COAD. **(B)** GO enrichment analysis of CTHRC1 in cellular components of COAD. **(C)** GO enrichment analysis of CTHRC1 in molecular function of COAD. **(D)** KEGG pathway enrichment analysis of CTHRC1 in COAD.

*CTHRC1* was co-expressed with *ROR2*, *VAPA*, *LY6E* and several collagen family proteins via GeneMANIA analysis (Figure 7). These associated molecules are mainly involved in collagen fibril, extracellular matrix/structure and external encapsulating structure organisations of biological processes; the collagen type I trimer, fibrillar collagen trimer and banded collagen fibril of cellular component construction; the platelet-derived growth factor binding, FFAT motif binding, extracellular matrix structural constituent conferring tensile strength and Wnt-protein binding of molecular function. Moreover, KEGG analysis using ShinyGO showed that *CTHRC1* and its related proteins were predominantly enriched in the signalling pathways of AGE-RAGE, Relaxin and Pl3K-Akt; the pathological processes of small cell lung cancer, amoebiasis and human papillomavirus infection; and the physiological functions of protein digestion and absorption, ECM-receptor interaction, focal adhesion and platelet activation (Figure 8).

## Discussion

Biomarkers are key tools for early diagnosis, prediction of survival and prognosis, and evaluation of treatment responses. Effective biomarkers benefit clinical decision-making and improve the patient’s survival rate and life quality (Ogunwobi et al., 2020). With the development of omics technology, many potential indicator genes have been screened for various diseases, such as adenomatous polyposis, transforming growth factor *β* and several tumour suppressors, which are widely used in the auxiliary diagnosis of tumorigenesis (Fodde et al., 2001; Gyorffy et al., 2013; Seoane and Gomis, 2017). However, the clinical application of these indicator genes is limited due to the lack of sufficient systematic research.

CTHRC1 is a glycosylated protein secreted during tissue repair, which functions by reducing the collagen matrix deposition, thereby promoting cell migration. The expression of *CTHRC1* under physiological conditions promotes wound healing; however, the pathological overexpression of *CTHRC1* promotes tumour cell growth and invasion (Cheng et al., 2019). Studies have shown that *CTHRC1* promotes colorectal cancer metastasis by inducing the Wnt/PCP signal transduction (Yang et al., 2015). In hepatocellular carcinoma, supressing *CTHRC1* expression can inhibit integrin *β*, and thereby inhibiting cell migration and invasion and inducing apoptosis (Zhou et al., 2019). Additionally, *CTHRC1* promotes the invasion of human epithelial ovarian cancer cells by activating the epidermal growth factor receptor signalling pathway (Ye et al., 2016). In short, the tumour promoting mechanism of *CTHRC1* involves multiple targets.

The occurrence and development of COAD have the common characteristics of tumorigenesis. It promotes the massive proliferation of cells by avoiding growth inhibitory factors and apoptosis-related genes and the production of tumour blood vessels to induce tumour development. Tumorigenesis mechanisms are also attributed to unstable gene expression, tumour microenvironment change, genetic diversity and inflammatory factor activation (Goubran et al., 2014). To understand the mechanism of *CTHRC1* in COAD and evaluate its potential as a diagnostic and prognostic indicator in patients with COAD, various online public databases were used to systematically investigate *CTHRC1* expression profiles in COAD tissue, its impact on patient survival and immunity, and its related functional pathways and associated proteins. The consistently high expression of *CTHRC1* in patients with COAD under different physiological and pathological states reflects its stability as a diagnostic indicator. Moreover, *CTHRC1* was highly expressed in at least 16 tumour types (Tang et al., 2006), which broadened the application of *CTHRC1* in early disease detection. Tumours usually have the characteristics of high metastasis tendency. Hence, patients aged 21–40 years and/or with Stage I COAD are more likely to obtain satisfying treatment responses; however, they are not easily diagnosed at the early stages. Notably, *CTHRC1* was obviously highly-expressed in these patients, which further highlights its value as an indicator gene. Further analysis of the prognostic value revealed that the characteristics of the high hazard ratio of *CTHRC1* expression and significant differences in prognostic survival indicate the superiority of *CTHRC1* as a prognostic biomarker, even the high mutation frequency would not offset its indicating effect on prognosis. Therefore, these data emphasise the high value of *CTHRC1* as a diagnostic and prognostic indicator for patients with COAD.

During tumorigenesis, due to the intervention of non-coding RNA, such as microRNA, the expression level of mRNA may be inconsistent with that of the associated protein (Macfarlane and Murphy, 2010). Taking this into account, the gene and protein levels were detected separately, revealing that in transcriptional and translational level, *CTHRC1* was both highly expressed in COAD tissues. Since the time-consuming, laborious and expensive method of protein level detection, the follow-up investigation is mainly based on the analysis of mRNA level, which ensures the effectiveness and improves the convenience of *CTHRC1* for future clinical research and application.

The response mechanism of the immune system in tumorigenesis has always been a popular research topic. The fluctuation of the tumour microenvironment is strongly related to the progress and treatment of the tumour. Understanding the tumour microenvironment provides an insight into tumour diagnosis, therapeutic targets and prognostic biomarkers (Goubran et al., 2014). The correlation analysis between *CTHRC1* level and B cell infiltration showed no significant correlation, which suggests that COAD avoided the immune effect of B cells. However, this observation needs further verification by deeper and profound studies. *CTHRC1* showed a close correlation (>0.5) with the infiltration levels of macrophages, neutrophils, dendritic cells and immune cell biomarker genes, which could be used as early screening targets for immunotherapy.

Through the enrichment analyses of GO and KEGG, the mechanism of *CTHRC1* in biological processes, not only its cellular components and molecular function were defined, but also the specific regulatory pathways (such as AGE-RAGE, Relaxin and Pl3K signalling pathways) and action targets (including protein digestion and absorption, ECM-receptor interaction, focal adhesion and platelet activation) were identified, most of which are classical regulatory models that promote tumorigenesis or are involved in tumour development (Feng et al., 2009; Bao et al., 2019; Waghela et al., 2021). Therefore, unravelling the tumour promoting mechanism of *CTHRC1*could narrow the scope of further research and drug screening.

The promoting effect of *CTHRC1* on tumour metastasis and proliferation could be considered a contributing factor to its abnormal high expression in various cancers including COAD. After systematic analysis, this study recommends *CTHRC1* as a biomarker gene for the early diagnosis and prognostic monitoring of COAD. This study aims to provide a base for future research, regarding the molecular mechanism and therapeutics development for COAD. However, verification of the clinical applications is still lacking although this study uses many databases for comprehensive analyses and comparison.

## Data Availability

The original contributions presented in the study are included in the article/Supplementary Material, further inquiries can be directed to the corresponding authors.
